# Zinc Deficiency Dermatitis in Cholestatic Extremely Premature Infants After a Nationwide Shortage of Injectable Zinc — Washington, DC, December 2012

**Published:** 2013-02-22

**Authors:** Scott A. Norton, Lamia Soghier, June Hatfield, Jeffrey Lapinski, Wanda D. Barfield

**Affiliations:** Dept of Dermatology; Dept of Neonatology, Children’s National Medical Center, Washington, DC; Div of Reproductive Health, National Center for Chronic Disease Prevention and Health Promotion, CDC

In mid-December 2012, three extremely premature infants with cholestasis in a neonatal intensive care unit (NICU) developed dermatitis in the diaper region, perioral erosions, and bullae on the dorsal surfaces of their hands and feet (Figure). The infants were similar in gestational age (23–24 weeks) and corrected postnatal age (33–38 weeks). All had severe cholestasis (direct bilirubin >3 mg/dL) and had received prolonged parenteral nutrition (PN). Each infant was in a private room and cared for by different nurses.

A search for environmental causes addressed infectious and toxic etiologies, medication reactions, use of new adhesives, and changes in PN. Searches for an infectious cause, including bacterial (wound, blood, urine, cerebrospinal fluid) and viral cultures, were negative. One infant treated empirically with intravenous antibiotics and acyclovir showed no improvement.

Recognition of the nationwide shortage of injectable zinc focused attention on the possibility of zinc deficiency. The hospital’s PN pharmacy exhausted its supply of injectable zinc on November 21, 2012, and the infants had not received zinc supplementation as of mid-December. Because other preparations of parenteral trace elements contain insufficient zinc to meet premature infants’ requirements and might cause trace element toxicity in cholestatic infants, no alternatives to the injectable zinc supplements were available.

The ranges of levels of plasma zinc (14–56 *μ*g/dL [normal: 70–120 *μ*g/dL]) and alkaline phosphatase, a zinc-dependent enzyme (32–62 U/L [normal: 150–420 U/L]), in the three infants were markedly low. Skin biopsy specimens from two of the infants showed findings consistent with zinc deficiency dermatitis. The fraternal twin of one of the infants received full-formula feedings and was clinically and biochemically unaffected. The infants’ skin lesions were managed with petrolatum dressings, and their PN was supplemented with zinc-containing enteral supplements. As their zinc levels improved, so did their skin lesions.

Zinc is an essential cofactor in approximately 300 enzyme-dependent processes. Fetal zinc accumulation via placental transport is maximal at 24–34 weeks of gestation. Extremely premature infants require 400 mg/kg per day because of negligible tissue stores of zinc, low albumin binding, increased catabolic state, and increased urinary zinc losses (1). Inadequate zinc supplementation leads to cutaneous changes, diarrhea, immunologic impairment, growth failure, and poor wound healing.

Because of the nationwide shortage of injectable zinc, other NICUs caring for PN-dependent, extremely premature, cholestatic infants might encounter similar cases. The two U.S. manufacturers of injectable zinc, Hospira (zinc chloride) and American Regent (zinc sulfate), have no available inventory. Hospira expects to resume production in March 2013 (2). The American Society for Parenteral and Enteral Nutrition provides recommendations for conserving and prioritizing trace element products in short supply (3). NICUs should monitor levels of zinc in infants at risk.

## Figures and Tables

**FIGURE:**
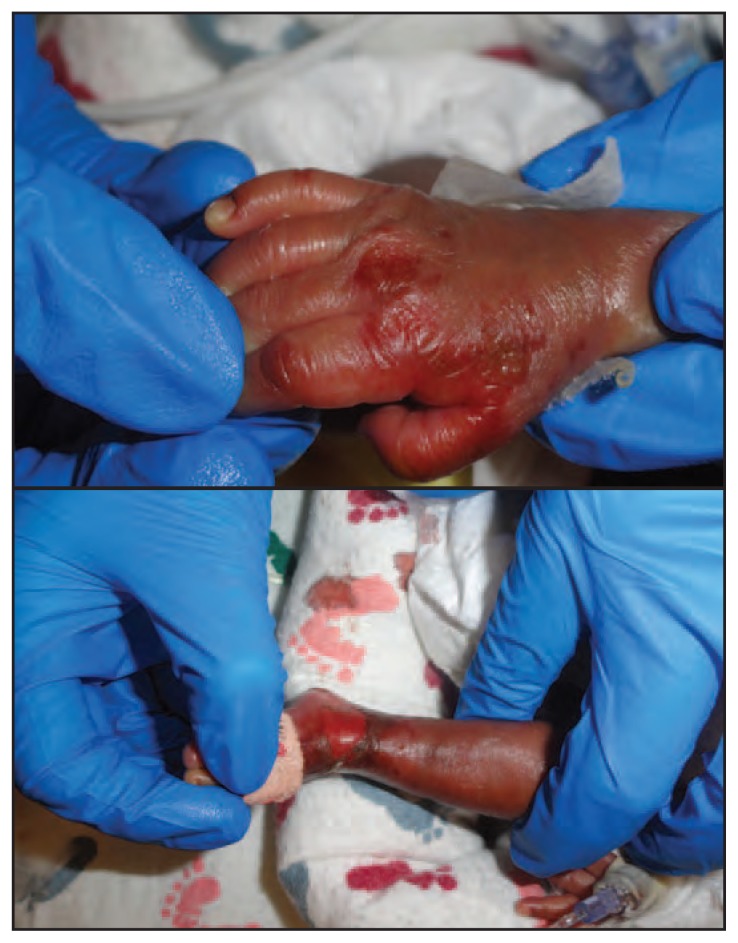
Zinc deficiency dermatitis manifesting as bullous and erosive lesions on the hands and feet of a newborn infant — Washington, DC, December 2012 Photo/S.A. Norton, Children’s National Medical Center
