# Erratum

**Published:** 2013-03-15

## Vol. 62, No. 7

In “Notes from the Field: Zinc Deficiency Dermatitis in Cholestatic Extremely Premature Infant After a Nationwide Shortage of Injectable Zinc — Washington, DC, December 2012,” an error occurred. On page 136, in the fifth paragraph, the third sentence should read, “Extremely premature infants require 400 ***μ*g/kg** per day because of negligible tissue stores of zinc, low albumin binding, increased catabolic state, and increased urinary zinc losses (*1*).”

